# Safety and feasibility of neoadjuvant chemotherapy as a surgical bridge for acute left-sided malignant colorectal obstruction: a retrospective study

**DOI:** 10.1186/s12885-022-09906-5

**Published:** 2022-07-21

**Authors:** Jiawei Zhang, Jiaxin Deng, Jiancong Hu, Qinghua Zhong, Juan Li, Mingli Su, Wei Liu, Miwei Lv, Tian Xu, Dezheng Lin, Xuefeng Guo

**Affiliations:** 1 Department of Endoscopic Surgery, Sun Yat-sen University Sixth Affiliated Hospital, No. 26 Yuancun Erheng Road, Guangzhou, 510655 China; 2 Guangdong Provincial Key Laboratory of Colorectal and Pelvic Floor Diseases, The Sixth Affiliated Hospital, Sun Yat-sen University, Guangzhou, China; 3 Xizang Minzu University, Xianyang, Shanxi China

**Keywords:** Left-sided malignant colorectal obstruction, Self-expandable metallic stent, Neoadjuvant chemotherapy, Stoma

## Abstract

**Background:**

For colorectal cancer, preoperative (neoadjuvant) chemotherapy is more effective than postoperative chemotherapy because it not only eradicates micrometastases more effectively but also reduces the risk of incomplete intraoperative resection and tumor cell shedding. For the treatment of acute left-sided malignant colorectal obstruction, colorectal stents as well as stoma are being used to relieve the obstructive colorectal cancer, and as a bridge to surgery, allowing easy mobilization and resection of the colon. Neoadjuvant chemotherapy combined with self-expandable metal stents (SEMS) or neoadjuvant chemotherapy combined with decompressing stoma (DS) can be used as a bridge to elective surgery (BTS) as an alternative to emergency surgery in patients with acute left-sided malignant colorectal obstruction, but its benefit is uncertain. The purpose of this study was to evaluate the safety and feasibility of neoadjuvant chemotherapy as a bridge to surgery in the treatment of acute left-sided malignant colorectal obstruction.

**Methods:**

Data from patients who were admitted with acute left-sided malignant colorectal obstruction between January 2012 and December 2020 were retrospectively reviewed, and patients with gastrointestinal perforation or peritonitis were excluded. We performed one-to-two propensity score matching to compare the stoma requirement, postoperative complications, and other short-term oncological outcomes between the neoadjuvant chemotherapy group and surgery group.

**Results:**

There were no differences in intraoperative blood loss, operative time, one-year postoperative mortality, and postoperative tumor markers between the two groups. The 1-year recurrence-free survival (RFS) rates of neoadjuvant chemotherapy group and surgery group were 96.8 and 91.3% (*p =* 0.562). The neoadjuvant chemotherapy group was able to reduce stoma rate 1 year after surgery (*p =* 0.047). Besides, the neoadjuvant group significantly reduced postoperative bowel function time (*p <* 0.001), postoperative hospital stay (*p <* 0.001), total hospital stay (*p =* 0.002), postoperative complications (*p =* 0.017), reduction in need to stay in the intensive care unit (ICU) (*p =* 0.042).

**Conclusions:**

Neoadjuvant chemotherapy as a bridge to elective surgery in patients with acute left-sided malignant colorectal obstruction is safe and has many advantages. Prospective multicenter studies with large samples are needed to further evaluate the feasibility of neoadjuvant chemotherapy.

## Introduction

Colorectal cancer has become the third most common malignancy and the fourth leading cause of cancer death [1, 2]. Acute obstruction occurs in approximately 8-29% of patients with colorectal cancer [3, 4]. Among them, the incidence of bowel obstruction with left colorectal cancer is higher. Intestinal obstruction can cause colonic edema resulting in a poorer general condition of patients, which could lead to a series of serious complications such as water and electrolyte imbalance, acid-base imbalance, peritonitis, intestinal perforation, and septic shock [5].

Obstruction relief and oncological clearance remain the focus in the treatment of colorectal cancer obstruction. Although emergency surgery is effective to relieve obstruction, it may increase the chance of stoma and has higher mortality and morbidity than elective surgery [6, 7]. About 60% of patients have a permanent stoma after surgery, which reduces quality of life. Anxiety and embarrassment over a stoma may lead to an alteration in lifestyle (including the ability to find a job or the desire to travel), body image (diet and clothing), and behaviors toward families and friends. Whether the stoma could be closed in time is also a frequent concern of patients [8]. To avoid emergency surgery, a strategy of self-expandable metallic stent (SEMS) or decompressing stoma (DS) as a bridge to elective surgery (BTS) in patients with acute left-sided malignant colorectal obstruction has been taken into account by more and more doctors. Prevention of malnutrition by allowing sufficient time for elective surgery can improve the prognosis and tolerance of tumor resection surgery and give surgeons more time to make a comprehensive and detailed preoperative evaluation. BTS can be accomplished by SEMS placement or DS construction [9–11]. SEMS was introduced in the 1990s for the palliative treatment of malignant colorectal obstruction [12, 13]. Later, SEMS began to be used to bridge acute left-sided malignant colorectal obstruction to elective surgery. Numerous published randomized controlled trials and some meta-analyses have shown that SEMS as a surgical bridge is safe and feasible in acute left-sided malignant colorectal obstruction [7, 14]. In addition, the European Society of Gastrointestinal Endoscopy (ESGE) guidelines recommend SEMS placement as an alternative to emergency resection [15]. DS construction can also be used as a BTS for surgery for acute left-sided malignant colorectal obstruction, a meta-analysis showed that patients treated with a colostomy followed by elective resection had significantly more primary anastomoses constructed and less stoma construction [16]. Neoadjuvant chemotherapy increases the opportunity of negative resection margins, early treatment of potential lymph node and/or distant micrometastases, and downstaging of colorectal cancer [17, 18]. Neoadjuvant chemotherapy may be used for acute left-sided malignant colorectal obstruction to increase the likelihood of resectability [19]. In addition, neoadjuvant chemotherapy is highly responsive to colorectal cancer due to its acceptable chemotherapy toxicity [20–22].

The ESGE recommends a time interval of approximately 2 weeks from the placement of a SEMS to elective resection [15]. After the creation of a DS, a bridging interval of 2-4 weeks is suggested [23]. Combining neoadjuvant chemotherapy with SEMS or DS as a BTS interval for elective surgery for acute left-sided malignant colorectal obstruction may help improve oncological outcomes [24]. However, few studies focus on this field. The purpose of this study is to review the short-term safety and feasibility of neoadjuvant chemotherapy with SEMS or DS as a surgical bridge for the treatment of acute left-sided malignant colorectal obstruction.

## Materials and methods

### Patients

This is a single-institution retrospective study of patients with acute left-sided malignant colorectal obstruction, treated with consecutive SEMS placement or decompression stoma and neoadjuvant before elective surgery in the Sixth Affiliated Hospital of Sun Yat-sen University from January 2012 to December 2020. We retrospectively collected clinicopathological characteristics of patients, including gender, age, location of obstructive lesions, clinical cancer stage, comorbidities, American Society of Anesthesiologists (ASA) classification, neoadjuvant chemotherapy regimens, preoperative carcinoembryonic antigen (CEA), preoperative albumin and hemoglobin, and postoperative CEA. Perioperative details including operation type, operation time, intraoperative blood loss, recovery time of bowel function, postoperative hospital stay, total hospital stay, ICU hospital stay, postoperative adjuvant chemotherapy, perioperative complications, and histopathological findings, as well as the presence of stoma, recurrence, and death 1 year after surgery were collected. The study was approved by the institutional ethics board of the Sixth Affiliated Hospital, Sun Yat-sen University (NO. 2022ZSLYEC-121). We confirm that we have obtained ethical approval to conduct the study as well as permission from the dataset, and the study was conducted in accordance with the provisions of the Declaration of Helsinki (as revised in Fortaleza, Brazil, October 2013). The obtained data were only collected and analyzed; however, detailed information was not released in public, and information confidentiality regulations were strictly adhered to.

### Definitions

Acute left colonic obstruction is diagnosed by clinical symptoms (bloating, pain, and inability to pass stool or gas), clinical examination, endoscopy, abdominal plain radiography, and abdominal computed tomography (CT). Left-sided colon cancer was defined as carcinoma of the splenic flexure and distal to the splenic flexure. Radical surgery was defined as complete resection of any measurable tumor, without involving resection of the margins. Bridging time was defined as from the date of stenting or stoma to the date of surgery. Technical success of self-expandable metal stent placement was defined as successful deployment of the SEMS through the obstructing lesion, radiographically confirmed stent expansion and clear visualization of the fecal passage. Clinical success was defined as significant colonic decompression on abdominal radiograph or CT, resolution of obstructive symptoms, and absence of SEMS-related complications. The recovery time of bowel function is the time from surgery to the first release of gas or defecation. The postoperative hospital stay is the time from a resection of colorectal tumor to discharge from hospital. The death of colorectal cancer 1 year after the operation is the death of patients within 1 year after the radical resection of colorectal cancer. Local recurrence was defined as colorectal, anastomotic, regional lymph node related, mesenteric, or peritoneal recurrence. Whether the patients died or recur 1 year after the operation was tracked according to the medical record system. Some patients were deleted due to the loss of follow-up in the database.

### Inclusion and exclusion criteria

Inclusion criteria included patients with colorectal obstruction due to left-sided malignant colorectal cancer and received curative treatment of surgery. Exclusion criteria were bowel ischemia, suspected or imminent perforation, contraindication to endoscopic therapy, obstruction caused by non-colonic malignancy or benign disease, without tumor resection, history of colectomy, and SEMS implantation in other hospitals.

**Fig. 1:**
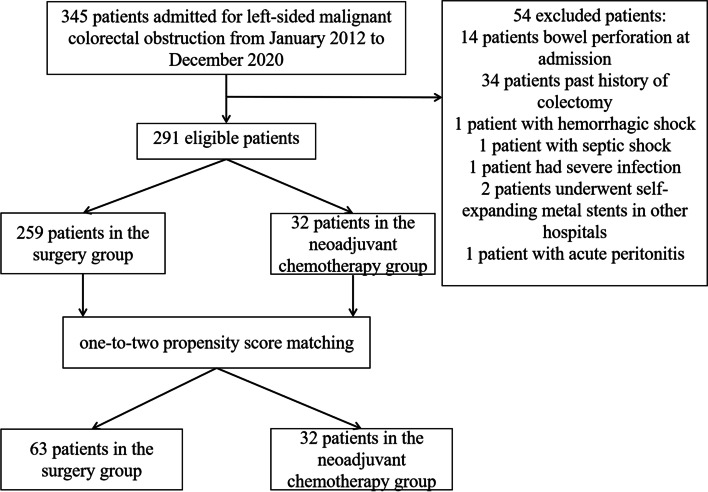
The flowchart of patient allocation

A flowchart of patient allocation is shown in Fig. 1. After application of inclusion and exclusion criteria, 291 patients were included in the study. After detailed exclusion, 54 patients were excluded. Reasons for exclusion were patients with bowel perforation (*n =* 14), previous left-sided colorectal cancer resection (*n =* 34), hemorrhagic shock (*n =* 1), septic shock (*n =* 1), severe infection (*n =* 1), underwent SEMS in other hospitals (*n =* 2), and patient with acute peritonitis (*n =* 1). Using one-to-two propensity score matching, 32 patients in the neoadjuvant chemotherapy group were matched to 63 patients in the surgery group (Fig. 1). In the surgery group, of which 41 patients underwent emergency surgery directly, and 12 patients performed an elective surgery after SEMS insertion. Besides, in the neoadjuvant chemotherapy group, of which 9 patients underwent DS and neoadjuvant chemotherapy as BTS to perform elective surgery, and 23 patients underwent SEMS and neoadjuvant chemotherapy as BTS to perform elective surgery.

### Treatment

The neoadjuvant chemotherapy group consisted of SEMS and DS as BTS to perform elective surgery, which all of them accepted neoadjuvant chemotherapy before elective surgery. All patients underwent standard colectomy and regional lymphadenectomy. The surgical approaches, operation methods, and the range of resection were determined by surgeons based on the tumor location, tumor stage, and patients’ general condition. Depending on the location of obstructive lesion and presence of edematous bowels, left colectomy, anterior resection, low anterior resection, subtotal colectomy, abdominoperineal resection, and Hartmann operation were performed by well-experienced colorectal surgeons in our single center. All SEMS placements were performed by well-experienced endoscopists using a WallFlex colonic stent (Boston Scientific) or Evolution colonic stent (Cook Ireland Limited). The placement of SEMS includes interventional placement and endoscopic placement. For interventional placement, the patient was placed on the DSA operating bed supine, the perineum was routinely sterilized and draped, a 5F-DAV catheter was placed through the anus, and the catheter-guided angiography showed the site of lesion obstruction, and a guide wire was inserted through the catheter. After reaching the distal end, transcatheter angiography showed obvious expansion and gas accumulation in the obstructed part of the lesion. A rigid guide wire was placed through the catheter to the lesion obstruction site, and the obstruction site and length were determined again. Endoscopic SEMS implantation: intravenous injection of midazolam, phloroglucinol and dezocine, observation of the guide wire placed in the stenotic site under DSA, and the smooth passage of the guide wire. In the stenotic part, a SEMS was introduced under direct vision. Patients’ vital signs and clinical status were monitored throughout the perioperative period. After SEMS insertion, improvement of the obstruction was monitored by the patients’ abdominal symptoms and abdominal X-ray. DS procedures include ileostomy and transverse colostomy. Neoadjuvant chemotherapy was administered after SEMS insertion or stoma decompression.

In this study, 32 patients received neoadjuvant chemotherapy, of which 23 patients received a median of 4 courses (IQR, 3-6 courses) of FOLFOX (oxaliplatin 85 mg/m2; folinic acid 400 mg/m2, followed by 5-FU, as a 400 mg/m2 intravenous bolus then a 2400 mg/m2 infusion over 46 h, days 1 and 2 of a 14-day cycle), 7 patients received a median of 8 courses (IQR, 6-10 courses) of FOLFOXIRI (oxaliplatin 85 mg/m^2^ dissolved in 500 ml of 5% glucose solution for intravenous infusion for 2 hours; irinotecan 150-165 mg/m^2^ dissolved in 250 ml of 0.9% sodium chloride for intravenous infusion for 90 minutes; followed by intravenous infusion inject folinic acid 400 mg/m^2^ for 2 h, on the first day; 5-FU 2800 mg/m^2^, continuous intravenous infusion over 48 h; once every 2 weeks), 2 patients received a median of 2 courses (IQR, 2-2 courses) of XELOX (intravenous infusion of oxaliplatin 130 mg/m^2^ on day 1, oral capecitabine tablets 1000 mg/m^2^ from day 1 to day 14; rest for 1 week, as a complete cycle, continuous 2 cycles). Adverse events (mainly grade 1 and 2) occurred in 11 (34.4%) patients. The most common toxicities were leucopenia (12.5%), fatigue (9.4%), thrombocytopenia (3.1%), nausea and vomit (3.1%), and diarrhea (3.1%), seen in Table 1.

**Table 1:** Toxicities of neoadjuvant chemotherapy (*n =* 11)

Variables	Degree 1	Degree 2	Degree 3	Degree 4
Leukopenia	3	1		
Thrombocytopenia		1		
Neutropenia				1
Nausea and vomit	1			
Diarrhea		1		
Fatigue	3			

### Study outcomes

The primary outcome of this study was short-term clinical outcomes and the result of whether there is a stoma 1 year after the operation. Short-term clinical outcomes included postoperative complications, postoperative hospital stay, ICU treatment, and recovery of bowel function. Secondary outcomes included operative time, intraoperative blood loss, total hospital stay, and postoperative carcinoembryonic antigen, one-year postoperative mortality, and one-year locoregional recurrence rate.

### Statistical analysis

One-to-two propensity score matching was performed without replacement. Propensity scores were estimated using a generalized linear model based on gender, age, whether the tumor has distant metastasis, and body mass index. Categorical data were evaluated using either the Chi square or Fisher exact tests, whereas numerical data were evaluated using the Student’s t test or Mann–Whitney U test. Numerical variables were dichotomized according to clinical importance or the median value of each variable for cut-off. All *p* values were two-sided, and *p <* 0.05 was considered statistically significant.

## Results

### Baseline characteristics

In this study, a total of 291 patients who diagnosis with acute left-sided malignant colorectal obstruction between January 2012 to December 2020 were enrolled and 95 patients were finally included after propensity score matching. Their basic characteristics are shown in Table 2. There were no differences in characteristics between the two groups in terms of age, gender, tumor site, hemoglobin, albumin, ASA status, and preoperative tumor markers. That means demographic and clinical characteristics were balanced between two groups at baseline. Compared with the neoadjuvant chemotherapy group, there was no significant difference of hemoglobin and albumin in the surgery group before colorectal cancer resection (Hemoglobin: 119.0 (g/L) [IQR, 100.0-130.0 (g/L)] vs. 120.0 (g/L) [IQR, 88.8-130.8 (g/L)], *p =* 0.774; Albumin: 37.50 (g/L) [IQR, 34.70-40.50 (g/L)] vs. 37.05 (g/L) [IQR,33.53-39.45 (g/L)], *p =* 0.529), which means there is no significant difference in nutritional status between the two groups (Table 2).

**Table 2:** Demographics and baseline characteristics of patients

	Surgery* (*n =* 63)	Neoadjuvant chemotherapy* (*n =* 32)	*P* value
Age (year, mean ± SD)	55.75 ± 14.34	56.47 ± 14.16	0.816
BMI, kg/m^2^ (mean ± SD)	21.72 ± 3.50	21.54 ± 3.07	0.804
Gender, N%			0.838
Male	40 (63.5)	21 (65.6)	
Female	23 (36.5)	11 (34.4)	
Hemoglobin (g/L) median, (IQR)	119.0 (100.0-130.0)	120.0 (88.8-130.8)	0.774
Albumin (g/L) median, (IQR)	37.50 (34.70-40.50)	37.05 (33.53-39.45)	0.529
Tumor location, N%			0.224
Splenic flexure	12 (19.0)	3 (9.4)	
Descending	8 (12.7)	5 (15.6)	
Sigmoid	32 (50.8)	13 (40.6)	
Rectum	11 (17.5)	11 (34.4)	
Preoperative CEA, N%			0.114
Normal	23 (39.0)	18 (56.3)	
Elevated	36 (61.0)	14 (43.8)	
ASA class, N%			0.352
I	38 (60.3)	15 (46.9)	
II	24 (38.1)	17 (53.1)	
III	1 (1.6)	0 (0.0)	
IV	0 (0)	0 (0)	
pT status, N%			0.018
T1	0 (0)	0 (0)	
T2	1 (1.6)	2 (6.3)	
T3	29 (46.0)	22 (68.8)	
T4	33 (52.4)	8 (25.0)	
pN stage, N%			0.791
N0	23 (36.5)	14 (43.8)	
N1	24 (38.1)	9 (28.1)	
N2	11 (17.5)	7 (21.9)	

*BMI*: Body mass index, *ASA*: American Society of Anesthesiologists, *CEA: C*arcino-embryonic antigen, *Surgery: Immediate Emergency Colorectal Resection; *Neoadjuvant chemotherapy: Immediate SEMS or Decompressing Stoma followed by Neoadjuvant chemotherapy before Elective Colorectal Resection

### Surgical outcomes

**Fig. 2:**
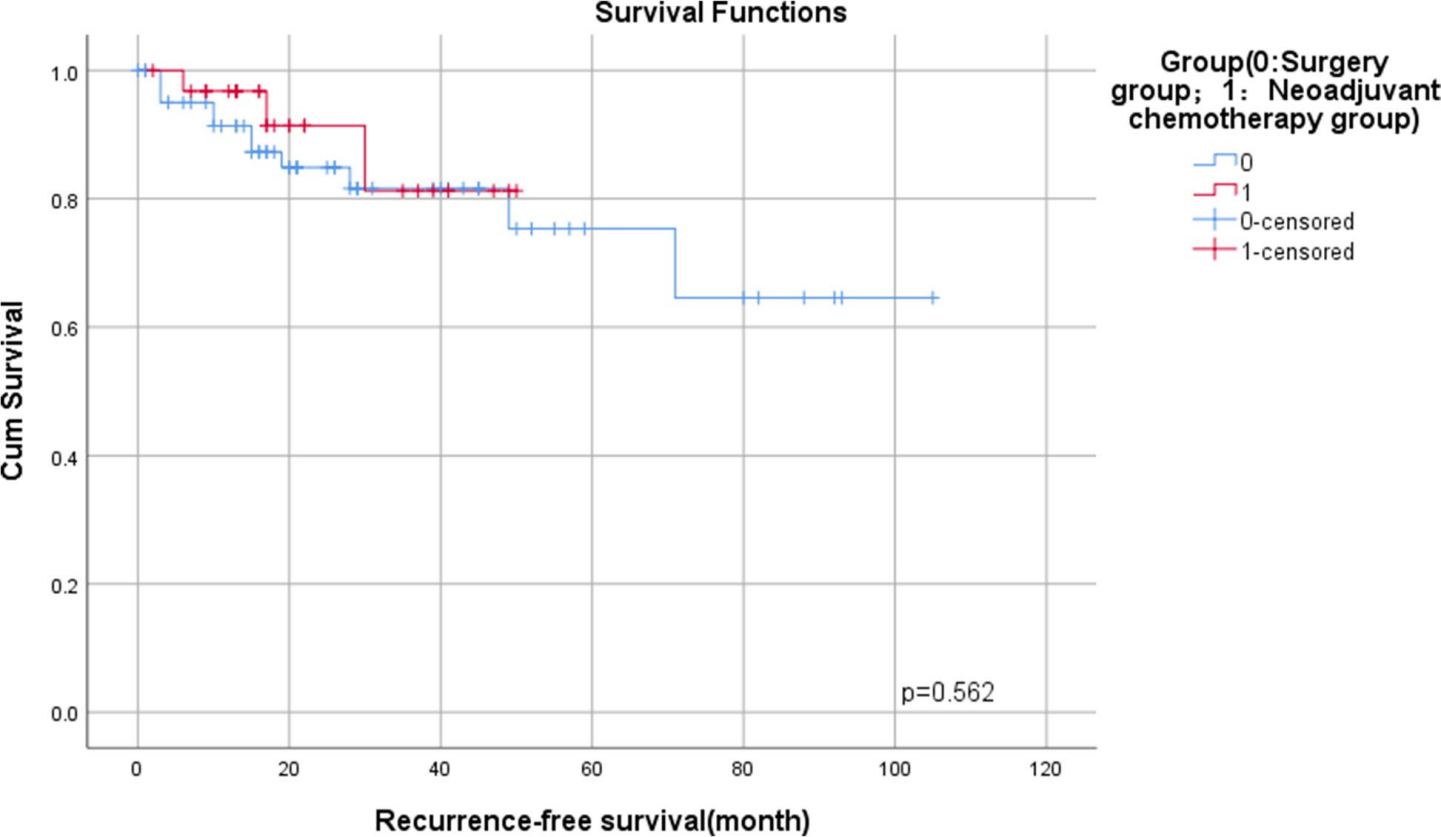
Comparison of the 1-year DFS rates between the surgery and neoadjuvant chemotherapy groups

Compared with the surgery group, there was a significant difference in the recovery time of bowel function in the neoadjuvant chemotherapy group (5.00d [IQR, 3.00-6.00d] vs. 3.00d [IQR, 2.00-3.00d], *p <* 0.001). The postoperative hospital stay and total hospital stay in the neoadjuvant chemotherapy group was significantly shorter than the surgery group respectively (13.00d [IQR, 10.00-19.00d] vs. 8.00d [IQR, 7.25-11.75d], *p <* 0.001; 20.00d [IQR, 16.00-25.00d] vs. 15.50d [IQR, 13.00-18.75d], *p =* 0.002). The operative time in the surgery group was slightly longer than that of the neoadjuvant chemotherapy group (230.00 min [IQR, 180.00-300.00 min] vs. 213.00 min [IQR, 188.50-270.00 min], *p =* 0.428). The stoma rate in the neoadjuvant chemotherapy group was significantly lower than that in the surgery group at 1 year after surgery (9.4% vs. 17.7%, *p =* 0.047). The surgery group had more local tumor recurrence 1 year after surgery than the neoadjuvant chemotherapy group (7.9% vs. 3.1%, *p =* 0.660). Postoperative mortality was higher in the surgery group (17.7% vs. 9.4%, *p =* 0.660). The 1-year recurrence-free survival (RFS) rates of the surgery and neoadjuvant chemotherapy group were 91.3 and 96.8%, *p =* 0.562 (Fig. 2).

There were no differences in intraoperative blood loss, operative time, one-year postoperative locoregional recurrence, one-year postoperative mortality, and postoperative tumor markers between the two groups (Table 3).

**Table 3:** Early Surgical outcomes

	Surgery* (*n =* 63)	Neoadjuvant chemotherapy*(*n =* 32)	*P* value
Postoperative CEA, N%			0.692
Normal	33 (63.5)	21 (67.7)	
Elevated	19 (36.5)	10 (32.3)	
Postoperative complication, N%			**0.001**
No	46 (73.0)	32 (100.0)	
Yes	17 (27.0)	0 (0.0)	
ICU, N%			**0.042**
No	53 (84.1)	32 (100.0)	
Yes	10 (15.9)	0 (0.0)	
Stoma, N%			0.198
No	21 (33.3)	15 (46.9)	
Yes	42 (66.7)	17 (53.1)	
1-year locoregional recurrence, N%			0.660
No	58 (92.1)	31 (96.9)	
Yes	5 (7.9)	1 (3.1)	
1-y Mortality, N%			0.439
No	51 (82.3)	29 (90.6)	
Yes	11 (17.7)	3 (9.4)	
1-y with stoma, N%			**0.047**
No	46 (73.0)	29 (90.6)	
Yes	17 (27.0)	3 (9.4)	
Postoperative bowel function (days), median, (IQR)	5.00 (3.00-6.00)	3.00 (2.00-3.00)	**< 0.001**
Postoperative hospital stay (days), median, (IQR)	13.00 (10.00-19.00)	8.00 (7.25-11.75)	**< 0.001**
Total Hospital stay (days), median, (IQR)	20.00 (16.00-25.00)	15.50 (13.00-18.75)	**0.002**
Surgery time (min), median, (IQR)	230.00 (180.00-300.00)	213.00 (188.50-270.00)	0.428
Intraoperative blood loss (ml), median, (IQR)	100.0 (50.0-200.0)	100.00 (50.00-187.50)	0.209

*Surgery: Immediate Emergency Colorectal Resection*;*

*Neoadjuvant chemotherapy: Immediate SEMS or Decompressing Stoma followed by Neoadjuvant chemotherapy before Elective Colorectal Resection

### Postoperative complication

For postoperative complications, the total number of complications was greater in the surgery group in the neoadjuvant chemotherapy group (27.0% vs 0.0%, *p =* 0.001).

In the surgery group, the most common complication was anastomotic leakage (7 cases, 41.2%), followed by abdominal wound infection (3 cases, 17.6%), lung infection (2 cases, 11.8%), abdominal infection (2 cases, 11.8%), organ failure (1 case, 5.9%), diarrhea (1 case, 5.9%), and urinary tract infection (1 case, 5.9%), seen in Table 4.

**Table 4:** Details of all complications after surgery

Postoperative complications	Surgery* (*n =* 17)	Neoadjuvant chemotherapy* (*n =* 0)
Anastomotic leakage	7	0
Abdominal infection	2	0
Organ failure	1	0
Diarrhea	1	0
Iung infection	2	0
Urinary tract infection	1	0
Abdominal wound infection	3	0

*Surgery: Immediate Emergency Colorectal Resection*;*

*Neoadjuvant chemotherapy: Immediate SEMS or Decompressing Stoma followed by Neoadjuvant chemotherapy before Elective Colorectal Resection

## Discussion

Treatment decision-making for acute left-sided malignant colorectal obstruction remains a challenging issue, and the management of non-metastatic acute left-sided malignant colorectal obstruction includes emergency primary surgical resection with SEMS or DS is a surgical bridge to select a strategy for transition to elective surgery [25]. For the clinical application of SEMS placement, Lamazza A et al. [26, 27]. reported that SEMS placement and decompressing stoma represent an important initial treatment approach for patients with obstructive colorectal cancer. SEMS placement and DS as a bridge to elective surgery offer many theoretical advantages, not only to correct problems such as fluid-electrolyte disturbances and cardiopulmonary function, but also to improve nutritional status, which potentially could transform an urgent clinical situation into an elective situation. With regard to the treatment strategies for acute malignant intestinal obstruction, Lamazza A et al. also reported that it should be based on a careful analysis of the different risk factors. The focus of treatment decisions is to relieve the obstruction while the main goal is to return to the desired tumor therapy as soon as possible. Neoadjuvant chemotherapy is effective and safe for non-metastatic left-sided malignant colorectal cancer [18, 20, 21, 24]. Numerous studies have shown that BTS can reduce operative mortality and improve surgical outcomes in acute left-sided malignant colorectal obstruction [9–11, 28]. A systematic review and meta-analysis showed that a total of 3894 patients were included. The blood loss during stent placement was less (*p <* 0.00001), the one-stage anastomosis rate was higher (RR1.25, *p <* 0.00001), the 30-day mortality rate was lower (RR0.65, *p <* 0.01), and the total complication rate was higher (RR0.65, *p <* 0.0001). However, in some cases we have no choice but to perform a brief colostomy. Incontinence manifestations and low rectal cancer 5-10 cm above the anal verge are contraindications for SEMS [29]. Severe abdominal pain with impending perforation and unstable vital signs (including hypotension, tachycardia, and tachypnea) are contraindications to SEMS placement. A retrospective study reported that a treatment of DS construction showed acceptable complication rates and feasible outcomes compared with SEMS placement. Considering the advantages and disadvantages of both methods, an individualized approach is necessary [30]. The optimal timing of elective surgery after stenting remains controversial. ESGE guidelines recommend a 2-week interval to allow complete decompression of the obstructed colon and resolution of tissue edema. This also provides an opportunity to optimize the patient’s nutritional status before starting the final surgery. It should be noted that, the edema of the bowel wall proximal to the obstruction persisted and the stoma rate was high when operated within 2 weeks of SEMS placement or decompressing stoma. Hooft et al. [28]. reported an interval of 5-14 days in a multicenter randomized controlled trial and found that the risk of anastomotic leakage increased due to insufficient intestinal decompression and recovery of systemic status when the interval between SEMS and surgery was short. Ormando et al. [31]. reported that a short interval from SEMS placement to surgery is an independent predictor of postoperative complications in patients undergoing elective surgery in a BTS setting. Besides, malignant colorectal obstruction is often in the advanced stage, which will occur micrometastases such as vascular/lymphatic invasion and nerve invasion. These might be the reasons for some patients received SEMS or DS followed by chemotherapy and finally elective surgery. Based on the above background and ambiguity of guidelines, surgeons would make individualized treatment strategies accordingly. By doing this, one-stage anastomosis is considered safe without the need for a stoma. There are few studies on the interval between elective surgery after DS [23]. Combining neoadjuvant chemotherapy during this interval as a treatment decision is poorly studied.

At present, most studies use SEMS or DS as a BTS for the treatment of acute left-sided malignant colorectal obstruction, and this study is based on neoadjuvant chemotherapy combined with SEMS or DS was used as a surgical bridge for the treatment of acute left-sided malignant colorectal obstruction. Our study also showed that this treatment option is feasible and safe, and it does not increase postoperative complications. During the longer interval between neoadjuvant chemotherapy combined with SEMS or neoadjuvant chemotherapy combined with DS as a BTS, the patient’s physical condition has improved, and neoadjuvant chemotherapy may be beneficial to the tumor, so it may help to reduce postoperative complications. Compared with emergency primary resection, the total hospital stay is shorter, and postoperative bowel function recovery time is shorter, and shorter hospital stay. In our study, the median time interval between neoadjuvant chemotherapy and colorectal cancer resection was 98.00 days, IQR (60.50-124.50). There is no clear study on the interval between neoadjuvant chemotherapy and surgical resection of colorectal cancer. Compared with the emergency surgery group, the operation time of the neoadjuvant chemotherapy group was shorter, which may be related to the gastrointestinal decompression performed in the emergency surgery and the increased difficulty of the operation due to intestinal edema caused by intestinal obstruction. As for whether this protocol could reduce the stoma rate and improve the first-stage anastomosis rate or not, our study shows that the stoma rate after 1 year in the emergency group is higher than that in the neoadjuvant chemotherapy group (3 patients, 9.4% vs 11 patients, 17.7%, *p =* 0.047). This is very meaningful for deciding whether a patient need a permanent stoma or not. At present, most patients are concerned about the best time to close the stoma. Closing the stoma as soon as possible can improve the quality of life. There were no significant differences between the groups in terms of recurrence and/or mortality after 1 year, due to uncertain effects of SEMS on long-term oncological outcomes, which may be due to the concerns about tumor manipulation during insertion, guidewire perforation during stent placement, stent deployment forces, and eventual micro-perforation at the proximal and distal ends of the stent, which may lead to local and bloodstream spread of tumor cells [32]. For our study, compared with the surgery group, the 1-year recurrence-free survival (RFS) rate was higher (91.3%vs 96.8%, *p =* 0.562). For patients with obstructive colorectal cancer, postoperative chemotherapy is often delayed due to delay, treatment compliance and postoperative complications. Neoadjuvant chemotherapy might be somewhat more likely to eradicate micrometastatic disease than might chemotherapy delayed until after surgery. Neoadjuvant chemotherapy may reduce the potential stimulatory effects of surgery on occult disease and reduce tumor cell shedding during surgery. Neoadjuvant chemotherapy strategies may improve RFS and reduce the risk of local recurrence in obstructive colorectal cancer [33, 34]. Whether BTS combined with neoadjuvant chemotherapy can reduce the spread of tumor cells in the local and bloodstream during stent placement, further long-term follow-up and prospective studies with large samples are needed to draw conclusions.

The current study has several limitations. These factors include the retrospective design of the study, potential selection bias due to physician or institutional bias when selecting stents for patients with obstructive colon cancer, the lack of a standard SEMS protocol, and it is difficult for us to standardize patient selection and management during the study period protocol, and a single-center retrospective study. The follow-up period was too short to draw meaningful conclusions about the long-term oncological effects of this treatment regimen. Prospective multicenter studies with large samples are needed to compare this treatment with traditional SEMS and to determine long-term oncologic and patient outcomes.

## Conclusions

Neoadjuvant chemotherapy as a bridge to elective surgery in patients with acute left-sided malignant colorectal obstruction is safe and has many advantages. Prospective multicenter studies with large samples are needed to further evaluate the feasibility of neoadjuvant chemotherapy.

## Data Availability

The datasets of the current study are not publicly available due to our institution policy but are available from the corresponding author on reasonable request.

## Abbreviations

ICU: intensive care units
BTS: Bridge to elective surgery
DS: Decompressing stoma
SEMS: Self-expandable metal stent
ESGE: European society for gastrointestinal endoscopy
CT: Computed tomography
DSA: Digital subtraction angiography

## References

[1] Arnold M (2017). Global patterns and trends in colorectal cancer incidence and mortality. Gut.

[2] Lekun Fang ZYMZ (2021). Clinical characteristics and survival analysis of colorectal cancer in China a retrospective cohort study with 13,328 patients from southern China. Gastroenterology report.

[3] Winner M (2013). Incidence and predictors of bowel obstruction in elderly patients with stage IV colon cancer: a population-based cohort study. JAMA Surg.

[4] June Hsu SS (2019). Management of Malignant Large-Bowel Obstruction. Dis Colon Rectum.

[5] Benedix F (2010). Comparison of 17,641 patients with right- and left-sided colon cancer: differences in epidemiology, perioperative course, histology, and survival. Dis Colon Rectum.

[6] Ghazal AH (2013). Colonic endolumenal stenting devices and elective surgery versus emergency subtotal/total colectomy in the management of malignant obstructed left colon carcinoma. J Gastrointest Surg.

[7] Ho KS (2012). Endoscopic stenting and elective surgery versus emergency surgery for left-sided malignant colonic obstruction: a prospective randomized trial. Int J Color Dis.

[8] Breitenstein S (2007). Systematic evaluation of surgical strategies for acute malignant left-sided colonic obstruction. Br J Surg.

[9] Jain SR (2020). Comparison of colonic stents, stomas and resection for obstructive left colon cancer: a meta-analysis. Tech Coloproctol.

[10] Veld JV (2020). Comparison of decompressing stoma vs stent as a bridge to surgery for left-sided obstructive Colon Cancer. JAMA Surg.

[11] Amelung FJ (2016). Deviating colostomy construction versus stent placement as bridge to surgery for malignant left-sided colonic obstruction. Surg Endosc.

[12] Camúñez F (2000). Malignant colorectal obstruction treated by means of self-expanding metallic stents: effectiveness before surgery and in palliation. Radiology.

[13] WATT AM (2007). Self-expanding metallic stents for relieving malignant colorectal obstruction : a systematic review. Ann Surg.

[14] Pirlet IA (2011). Emergency preoperative stenting versus surgery for acute left-sided malignant colonic obstruction: a multicenter randomized controlled trial. Surg Endosc.

[15] van Hooft JE (2020). Self-expandable metal stents for obstructing colonic and extracolonic cancer: European Society of Gastrointestinal Endoscopy (ESGE) guideline - update 2020. Endoscopy.

[16] Amelung FJ (2015). Acute resection versus bridge to surgery with diverting colostomy for patients with acute malignant left sided colonic obstruction: systematic review and meta-analysis. Surg Oncol.

[17] Maas M (2010). Long-term outcome in patients with a pathological complete response after chemoradiation for rectal cancer: a pooled analysis of individual patient data. Lancet Oncol.

[18] Ielpo JAEP (2020). Neoadjuvant chemotherapy in locally advanced colon cancer a systematic review. Tech Coloproctol.

[19] Symonds LK, Cohen SA (2019). Use of perioperative chemotherapy in colorectal cancer metastatic to the liver. Gastroenterology report.

[20] Zhou H (2016). A pilot phase II study of neoadjuvant triplet chemotherapy regimen in patients with locally advanced resectable colon cancer. Chin J Cancer Res.

[21] Arredondo J (2017). Mid-term oncologic outcome of a novel approach for locally advanced colon cancer with neoadjuvant chemotherapy and surgery. Clin Transl Oncol.

[22] Arredondo J (2014). Tumor response assessment in locally advanced colon cancer after neoadjuvant chemotherapy. J Gastrointest Oncol.

[23] Veld JV (2021). Time interval between self-expandable metal stent placement or creation of a decompressing stoma and elective resection of leftsided obstructive colon cancer. Endoscopy.

[24] Han JG (2020). Efficacy and safety of self-expanding metallic stent placement followed by neoadjuvant chemotherapy and scheduled surgery for treatment of obstructing left-sided colonic cancer. BMC Cancer.

[25] Malakorn S (2018). Urgent Management of Obstructing Colorectal Cancer: divert, stent, or resect?. J Gastrointest Surg.

[26] Lamazza A (2015). Endoscopic placement of self-expanding stents in patients with symptomatic anastomotic leakage after colorectal resection for cancer: long-term results. Endoscopy.

[27] Lamazza A (2016). Endoscopic placement of self-expandable metallic stents for rectovaginal fistula after colorectal resection: a comparison with proximal diverting ileostomy alone. Surg Endosc.

[28] van Hooft JE (2011). Colonic stenting versus emergency surgery for acute left-sided malignant colonic obstruction: a multicentre randomised trial. Lancet Oncol.

[29] Tomiki Y (2004). Comparison of stent placement and colostomy as palliative treatment for inoperable malignant colorectal obstruction. Surg Endosc.

[30] Jung WB, Shin JY, Park JK (2020). Comparison of short-term outcome between diverting colostomy and colonic stent as a bridge to surgery for left colonic malignant obstruction. Medicine (Baltimore).

[31] Ormando VM (2019). Colonic stents for malignant bowel obstruction: current status and future prospects. Expert Rev Med Devices.

[32] Broholm M (2017). Delay of surgery after stent placement for resectable malignant colorectal obstruction is associated with higher risk of recurrence. Int J Color Dis.

[33] Cercek A (2018). Adoption of Total neoadjuvant therapy for locally advanced rectal Cancer. JAMA oncology.

[34] Liu S (2021). Total neoadjuvant therapy (TNT) versus standard neoadjuvant Chemoradiotherapy for locally advanced rectal Cancer: a systematic review and Meta-analysis. Oncologist.

